# Targeted Molecular Therapeutics for Parkinson's Disease: A Role for Antisense Oligonucleotides?

**DOI:** 10.1002/mds.29201

**Published:** 2022-08-29

**Authors:** Dunhui Li, Frank L. Mastaglia, Wai Yan Yau, Shengdi Chen, Steve D. Wilton, Patrick A. Akkari

**Affiliations:** ^1^ Perron Institute for Neurological and Translational Science The University of Western Australia Nedlands Australia; ^2^ Centre for Molecular Medicine and Innovative Therapeutics Murdoch University Murdoch Australia; ^3^ College of Nursing and Health Zhengzhou University Zhengzhou China; ^4^ Department of Neurology and Institute of Neurology Ruijin Hospital Affiliated to Shanghai Jiao Tong University School of Medicine Shanghai China; ^5^ Department of Neurology Duke University Durham North Carolina USA

Parkinson's disease (PD) is a prevalent neurodegenerative disorder characterized by marked heterogeneity in clinical symptoms and a complex genetic background. Although effective to varying degrees, symptomatic treatments, including dopaminergic medications and device‐assisted therapies, can improve the quality of life for people with PD but do not halt disease progression. Effective disease‐modifying therapies remain elusive. Discussions remain over whether PD is a single disease or an umbrella term for a group of disorders with common features.^1^ Rather than representing a singular heterogeneous disorder, an alternative view is that PD encompasses a group of distinct biological entities with overlapping clinical manifestations, but each having a unique molecular pathogenesis. Based on this view, it is proposed that developing targeted therapies for more homogenous subgroups of patients with shared molecular genetic features will be more likely to succeed and to translate into the clinic.^2^

The approvals by the US Food and Drug Administration (FDA) of antisense oligonucleotide (ASO) therapeutics to treat spinal muscular atrophy, Duchenne muscular dystrophy (DMD), and familial amyloid polyneuropathy and ongoing clinical trials in patients with Huntington's disease, Alzheimer's disease, and amyotrophic lateral sclerosis^3^ have highlighted the therapeutic potency of ASOs for inherited neuromuscular and neurodegenerative disorders.

In this article, we point out opportunities and prospects of ASO therapies targeting specific aspects of PD pathogenesis on a background of the current state of knowledge of the genetic underpinnings as they pertain to familial PD (fPD) and sporadic PD (sPD).

## Monogenic Influences in fPD and sPD

Although the entire PD genetic architecture is yet to be uncovered, variations in more than 20 genes have been found to be disease causing (Table 1). These gene mutations affect critical cellular pathways and are responsible for monogenic fPD. Mutations in *SNCA*, *LRRK2*, and *VPS35* are linked to dysfunction in autophagy‐lysosome pathways and cause autosomal dominant PD, while pathogenic variations in *PRKN*, *PINK1*, and *DJ‐1* are generally associated with impaired mitochondrial functions and contribute to autosomal recessive PD. Although monogenic PD represents only ~5% of all PD cases and causative mutations of each individual gene are even rarer, emerging evidence has shown that genes and molecular pathways identified in monogenic PD also play essential roles in sPD.^4^ Although rare high‐penetrance mutations in those genes confer significant pathogenic effects and are disease causing, common risk variants at these pleomorphic loci exert minor effects and increase the cumulative genetic risk of developing PD.^5^

**TABLE 1:** Monogenic Parkinson's disease and pathophysiological pathways implicated

PARK symbol	Gene	Inheritance	Phenotype	Mutation	Likely disease mechanism	Lewy bodies	Main molecular pathways involved
PARK 1 and 4	*SNCA*	Dominant	EOPD	Missense and CNV	Gain of function^a^	Yes	UPS and autophagy‐lysosomal pathway
PARK 2	*PRKN*	Recessive	EOPD	Missense or deletion	Loss of function	No	Mitochondrial maintenance and UPS
PARK 5	*UCHL1*	Dominant	Classical PD	Missense	Loss of function	Unknown	UPS
PARK 6	*PINK1*	Recessive	EOPD	Missense	Loss of function	One case	Mitochondrial maintenance
PARK 7	*DJ1*	Recessive	EOPD	Missense	Loss of function	Unknown	Oxidative stress; aberrant splicing
PARK 8	*LRRK2*	Dominant	Classical PD	Missense	Gain of function	Yes^b^	Autophagy‐lysosomal pathway
PARK 9	*ATP13A2*	Recessive	EOPD	Missense	Loss of function	Unknown	Autophagy‐lysosomal pathway
PARK 11	*GIGYF2*	Dominant	LOPD	Missense	Loss of function	Unknown	Insulin‐like growth factor signaling
PARK 13	*HTRA2*	Dominant	Classical PD	Missense	Undetermined	Unknown	Mitochondrial maintenance
PARK 14	*PLA2G6*	Recessive	EOPD or AOPD	Missense	Loss of function	Yes	Mitochondrial maintenance
PARK 15	*FBXO7*	Recessive	EOPD	Missense	Loss of function	Unknown	UPS and mitochondrial maintenance
PARK 17	*VPS35*	Dominant	Classical PD	Missense	Loss of function	Yes	Endosomal trafficking
PARK 18	*EIF4G1*	Dominant	Classical PD	Missense	Undetermined	Yes	Mitochondrial maintenance
PARK 19	*DNAJC6*	Recessive	EOPD	Missense	Loss of function	Unknown	Endosomal trafficking
PARK 20	*SYNJ1*	Recessive	EOPD	Missense	Loss of function	Unknown	Endosomal trafficking
PARK 21	*TMEM230*	Dominant	Classical PD	Missense	Loss of function	Yes	Vesicle secretion and retromer trafficking
PARK 23	*VPS13C*	Recessive	EOPD	Missense	Loss of function		
NA	*GBA*	Dominant/Recessive	NA	Missense	Loss of function^c^	Yes	Autophagy‐lysosomal pathway
NA	*POLG*	Dominant	Atypical PD	Missense	Loss of function	Unclear	Mitochondrial respiration
NA	*LRP10*	Dominant	LOPD	Missense	Loss of function	Yes	Lipid homeostasis

^a^ Gain of function is the prevailing hypothesis for SNCA. However, loss of function and haploinsufficiency of SNCA mutations were also demonstrated to contribute to disease progression in familial PD.^20^, ^21^ In addition, the biophysical transformation of α‐synuclein may lead to the loss of function of soluble and bioactive α‐synuclein.

^b^ Both gain of function and loss of function have been proposed to explain the role of GBA in PD.^22^

^c^ The presence of Lewy body is variable in patients with LRRK2 mutations; a proportion of patients with PD as a result of LRRK2 mutations display an absence of apparent Lewy pathology.

EOPD, early‐onset PD; UPS, ubiquitin‐proteasome system; AOPD, adult‐onset PD; PD, Parkinson’s disease; LOPD, late‐onset PD, NA, not applicable; CNV, copy number variation.

The last decade has witnessed significant advances in identifying single‐nucleotide polymorphisms and structural variants that confer PD risk and has shown that genetic variations contribute to at least 25% of the risk of developing PD.^5^, ^6^ A total of 90 independent risk‐associated single‐nucleotide polymorphisms were identified in a GWAS meta‐analysis from a European ancestry dataset.^6^ Most of those risk loci are in proximity to *SNCA*, *LRRK2*, *GBA*, or *VPS13C*,^6^ which alters the gene expression and affects associated pathophysiological pathways. For example, *SNCA* rs356219 is associated with an increased expression of the SNCA112 isoform that has a greater propensity to form SNCA aggregates,^7^, ^8^ while a T allele at rs76904798 elevates LRRK2 expression and increases PD risk.^9^ Although current focus is on common variants in *SNCA*, *LRRK2*, and *GBA*, efforts are being made to discover additional genetic risk loci in cohorts of a wider ancestral diversity. Findings from these studies are anticipated to provide novel genetic markers to stratify patients in clinical trials of targeted therapies.

## Genetic and/or Molecular Pathway–Targeted Precision Medicine for PD

According to the PD “reconstruction model” proposed by Espay et al,^10^ the term PD refers to a group of distinct but overlapping biological entities and allows for evaluating novel targeted therapies in genetically and/or molecularly defined PD subtypes, thereby reducing confounding effects of patient heterogeneity. A few clinical trials targeting SNCA, LRRK2, and GBA have been designed under this concept (Table 2). Although PD is well established as an α‐synucleinopathy, based on the SNCA gain‐of‐function (GOF) hypothesis,^11^ attempts have been made to suppress SNCA expression to reduce its pathological effects.^12^ However, the central role of SNCA in PD pathogenesis more generally appears controversial and has been challenged by observations including frequent post mortem findings of mixed‐protein aggregate pathology,^8^ as well as poor correlation between Lewy body pathology and clinical features and between Braak staging and cell loss in the substantia nigra.^13^, ^14^

**TABLE 2:** Genetic and/or molecular pathway–targeted therapies in clinical trials for Parkinson's disease

Target	Mechanism	Drug name	Modality	Targeting cohort	Status	ID
SNCA	Decrease α‐synuclein expression	PRX002	Biologic	Early PD	Phase II; active, not recruiting	NCT03100149
		BIIB054	Biologic	Early PD	Phase II; terminated	NCT03318523
		MEDI1341	Biologic	HVs	Phase I; completed	NCT03272165
				Mild PD	Phase I; active, not recruiting	NCT04449484
		PD01A	Biologic	Early PD	Phase I; completed	NCT02267434
	Decrease α‐synuclein aggregation	NPT200‐11	Small molecule	HVs	Phase I; completed	NCT02606682
		K0706	Small molecule	Early PD	Phase II; recruiting	NCT03655236
	Increase α‐synuclein degradation	Nilotinib	Small molecule	Mild PD	Phase II; unknown	NCT02954978
				Early/mild PD	Phase II; completed	NCT03205488
				PDD	Phase I; completed	NCT02281474
LRRK2	Inhibit LRRK2 activity	BIIB094	ASO	LRRK2‐PD	Phase I; recruiting	NCT03976349
		DNL201	Small molecule	LRRK2‐PD	Phase I; completed	NCT03710707
GBA	Modulate GCase activity	Ambroxol	Small molecule	GBA‐PD	Phase II; completed	NCT02941822
				PDD	Phase II; recruiting	NCT02914366
		RTB101	Small molecule	GBA‐PD	Phase II; terminated	NCT02906020
		PR001		GBA‐PD	Phase I/IIa; recruiting	NCT04127578

PD, Parkinson's disease; HV, healthy volunteer; PDD, patients with PD with dementia; LRRK2‐PD, PD with or without LRRK2 mutations; GBA‐PD, PD with or without GBA mutations; GCase, glucocerebrosidase, ASO, antisense oligonucleotides.

Recent studies have suggested that conformational and biophysical protein transformation may actually result in a loss‐of‐function (LOF) of the original natively folded soluble protein,^15^ indicating that SNCA aggregation may cause an LOF of its soluble and bioactive form. Along with this, post mortem studies have shown that soluble SNCA levels decrease in PD‐vulnerable brain regions,^16^, ^17^ with increasing accumulation of insoluble SNCA over the disease course.^17^ Thus, nonselectively clearing SNCA may fail to deliver therapeutic benefits and could even have deleterious effects and contribute to neurodegeneration, as has been observed in rodents and nonhuman primates.^18^, ^19^ Combinational therapies may therefore be needed to ensure a proper balance between removing insoluble SNCA aggregates while maintaining levels of the soluble protein.

Strategies to reduce SNCA levels may however still be appropriate for some forms of PD, including PARK 1 and 4, where there is an increased SNCA expression, or for individuals with *SNCA* risk variants that alter its expression, if not for general PD or familial forms without Lewy body pathology. Although in the situation where there is co‐presentation with other protein pathologies such as tau or amyloid‐ß, targeting SNCA expression might be suitable if SNCA is proven to have a primary role. Notwithstanding, the issue that remains particularly challenging and controversial is whether clearing already formed SNCA aggregates would have any effect on arresting the aggregation process once it has been initiated.

## ASOs and Mechanisms of Action

**FIG 1:**
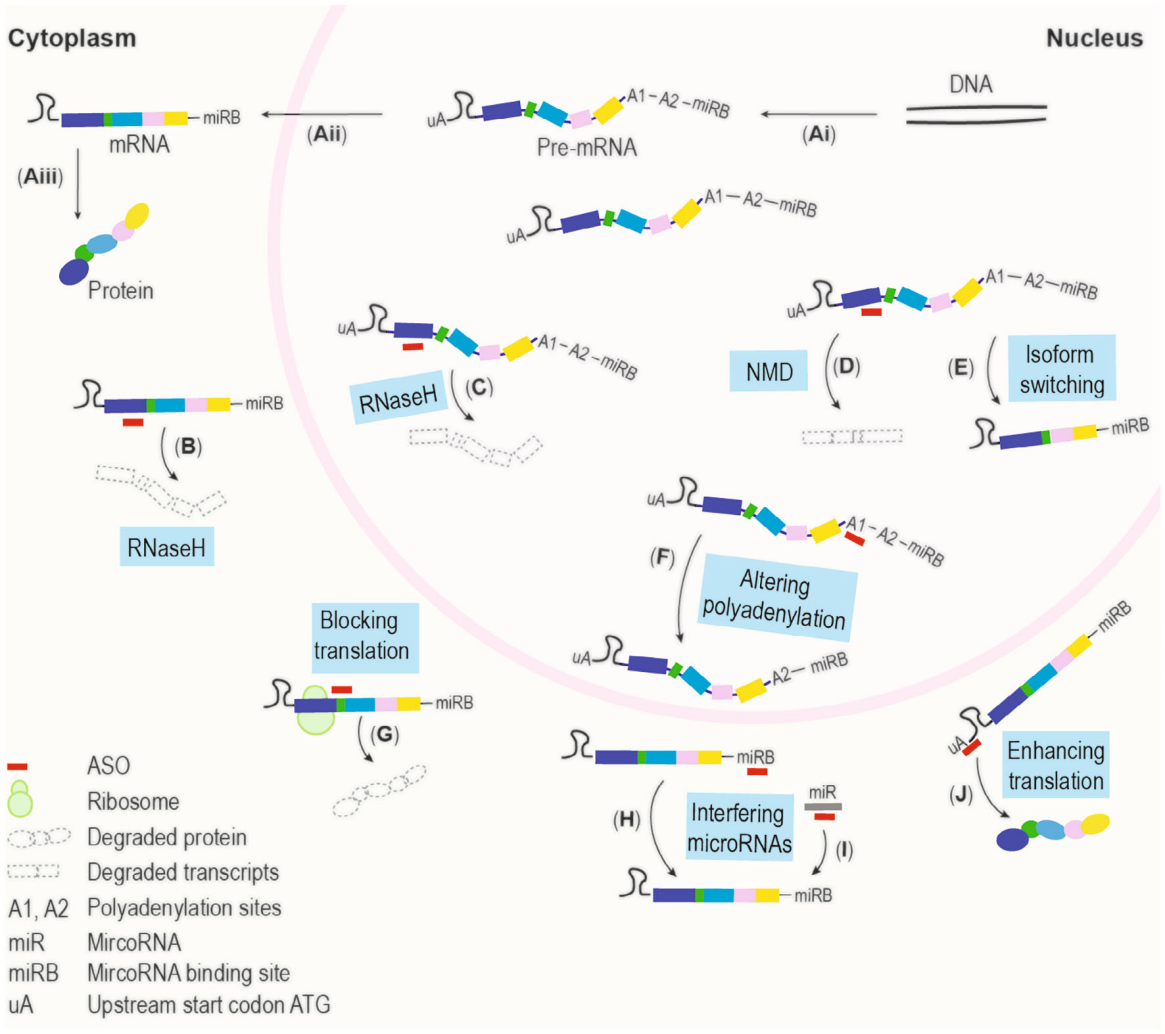
Major mechanisms of action of antisense oligonucleotides. General information flow of protein‐coding genes (**A**i–iii): DNA transcribed to pre‐mature RNA (pre‐mRNA) (i); pre‐mRNA processed to mature RNA (mRNA) (ii), which is translated to protein (iii); RNaseH‐inducing ASOs, normally gapmers, can degrade targeted transcripts in the cytoplasm (**B**) and nucleus (**C**). Splice‐modulating ASOs can skip an out‐of‐frame exon to create a premature stop codon, which induces non‐sense‐mediated decay (NMD) of a gene of interest (**D**). Splice‐switching ASOs can also generate an alternative transcript that could translate to a shorter and alternatively spliced protein isoform (**E**). The polyadenylation site of a targeted transcript can be altered by placing an ASO against either a proximal or distal polyadenylation signal, which could result in an unstable or a more stable transcript and the resulting reduced or increased protein production (**F**). ASOs can sterically block the translation initiation complex, block the ribosome, and inhibit mRNA translation (**G**). Through targeting microRNA binding sites (miRBs) (**H**) or specifically binding to microRNAs (miR) (**I**), ASOs can inhibit the functions of microRNAs to stabilize mRNA transcript to increase expression. By sterically blocking the upstream AUG (uA) in the upstream open reading frame or inhibitory motifs residing in the 5′ untranslated region, ASOs could enhance targeted transcript translation and increase the expression of the corresponding protein (**J**). [Color figure can be viewed at wileyonlinelibrary.com]

ASOs are short synthetic nucleic acid analogues that bind to target sequences through the Watson‐Crick base pairing, thereby specifically annealing to (pre‐)mature RNAs and inducing a variety of changes in RNA processing.^20^ The last two decades have witnessed impressive advances in ASO chemistries, allowing exploration of several different ASO mechanisms of action, including splice modulation, RNaseH‐mediated gene degradation, translation blocking, and enhancing translation by targeting regulatory motifs in the 3′ or 5′ untranslated regions.^20^, ^21^ In more general terms, the mechanisms of action can be divided into three categories: (1) downregulating gene expression to address GOF mutations (Fig. 1B,C,D,F,G), (2) correcting splice‐site mutations or redirecting expression of pathogenic isoforms (Fig. 1E), and (3) upregulating gene expression for LOF mutations (Fig. 1F,H,I,J).

## Potential Applications of ASO Therapies in PD

In addition to the application of other RNA‐based therapies, including small interfering RNAs (siRNAs), microRNAs, and aptamers in some PD subgroups, several ASO approaches have been evaluated in experimental models or can be considered for developing targeted therapeutics for fPD. Using ASO‐mediated gene downregulation, a compound to suppress LRRK2 expression has shown efficacy in a PD mouse model^1^, ^22^ and is currently undergoing evaluation in a phase I clinical trial (ClinicalTrials.gov: NCT03976349), with data suggesting that an allele‐specific approach may be needed. Allele‐specific approaches may also be applicable for GOF mutations in other PD genes, including the *SNCA* A53T mutation. Meanwhile, ASO‐mediated gene upregulation could potentially address haploinsufficiency caused by LOF mutations, as in the case of *GBA* or *SNCA* haploinsufficiency‐associated PD subtypes.^22^, ^23^ Such an approach could conceivably also be used to increase expression of native SNCA in combination with a therapy that clears its protein aggregates.

Through targeting RNAs,^3^, ^24^ ASOs are capable of targeting “undruggable” pathways for conventional small molecules and antibody therapies that require protein or protein‐active sites. It is known that less than ~1.5% of the human genome is protein coding, leaving a massive space for developing RNA‐targeting ASO therapeutics. Dysregulation of long noncoding RNAs and other cellular RNAs, including microRNAs, ribosomal RNAs, and small nuclear RNAs, have all been implicated in PD pathogenesis.^25^, ^26^ ASOs targeting noncoding RNAs have started to show promise, for example, an ASO‐based miR‐103a‐3p antagomir has shown efficacy in a PD mouse model.^27^

In addition, splice‐switching ASOs could have significant roles in correcting splice‐site mutations, which are enriched in sPD,^28^ and splicing defects in fPD, including PARK7 (*DJ‐1*).^28^ This approach could redirect expression of isoforms that have distinct pathogenic roles, as in the case of SNCA, or restore the expression of a deficient protein caused by frameshifting mutations, as in *PRKN* (PARK2).

### PRKN Mutations: An Example of Targeted ASO Therapy

PARK2 patients have a higher prevalence of atypical features such as dystonia and hyperreflexia and show an absence of Lewy body pathology,^29^, ^30^ thus being considered a distinct PD subgroup. Based on proof‐of‐concept genotype–phenotype correlations and protein function mapping studies,^31^, ^32^ an ASO‐mediated exon‐skipping strategy^33^ has successfully restored functional parkin protein expression in patient‐derived fibroblasts carrying amenable mutations. In a similar context, comparable exon‐skipping strategies have led to four US FDA‐approved ASO drugs targeting different disease‐causing dystrophin mutations to treat DMD.^20^

If validated in a precision animal model, or if as in cystic fibrosis animal models can be bypassed,^10^, ^34^ this PARK2 exon‐skipping approach has the potential to become a prototype for the application of targeted ASO therapeutics in PD. Although PARK2 patients amenable to this strategy make up only a small percentage of PD cases, their numbers collectively (around 3,824–8,754 cases in the United States and Europe) are not insubstantial when compared with other rare diseases. Because genetically or pathologically defined subtypes of a common disease can each be considered as being rare diseases in their own right, from the perspective of drug development,^35^ novel therapeutics for PARK2 could therefore be regarded as orphan drugs. This may bring greater efficiency in drug translation as proposed in the 2017 *FDA Orphan Drug Modernization Plan*.^36^

## Conclusions and Future Directions

Although the number of patients with PD amenable to gene‐specific or mutation‐specific ASO therapies is currently small due to the rarity of mutations in those causative genes, when similar antisense strategies are developed for other mutations or in other genes, this approach is likely to become relevant for a significantly larger number of patients. This has already occurred in other diseases, including DMD, where the successive FDA approvals of four exon‐skipping ASOs has increased the proportion of treatable DMD patients from 13% in 2016 to 30% in 2021. However, significant challenges still remain in fully elucidating the functional genomics of PD subtypes and unraveling the disease pathophysiology. Meanwhile, reliable biomarkers are still under exploration for subtyping PD and for monitoring disease progression and evaluating response to therapeutic interventions. Pathway‐specific biomarkers, such as the SNCA radiotracer^37^ for *SNCA*‐related PD and mitophagy pathway biomarkers for PARK2‐associated patients, might provide opportunities to monitor and evaluate the disease‐modifying effects of antisense therapeutics.

Before any treatment reaches the clinic, we must consider safety implications and the cost of any new therapeutic compared with current affordable and relatively stable treatments. Safety concerns over ASOs originate from backbone/chemical modification‐related toxicities^20^ and sequence‐specific off‐target effects^38^; however, these could be minimized or avoided by using ASO chemistries known to be clinically safe and by rational sequence design and in silico screening. We should not necessarily be dissuaded by the current annual cost of ~$300,000 per patient for ASOs to treat DMD when we consider that the cost of penicillin in the 1940s was $20 per 100,000 units, while it is now only $0.013 per 100,000 units.^39^ It is expected that with improvements in ASO chemistry and synthesis, and the increasing demand for ASO therapies, there will be a substantial cost reduction for ASOs with time. In addition, future progress is also needed to address current limitations and challenges in ASO technology, including the administration route, stability of ASOs in the bloodstream and cerebrospinal fluid, and the blood–brain barrier permeability of ASOs for central nervous system indications.^38^ It is likely that advances in ASO chemistry and delivery systems, including ASO‐peptide/antibody/nanoparticle conjugates, will enable less frequent dosing and more convenient ASO administration protocols.

Referring to the success of making levodopa, the most challenging compound to control therapeutically because of its short biological half‐life and “physicochemical cussedness,” into a standard symptomatic treatment for PD,^40^ we believe it is reasonable to forecast the success of developing ASOs as effective PD therapies.

## Author Roles

D.L. conceived and wrote the manuscript. D.L. and F.L.M. structured the paper. F.L.M., S.D.W., W.Y.Y., S.C., and P.A.A. reviewed and edited the manuscript. All authors read and approved the manuscript.

## Financial Disclosures

D.L. receives salary support from the philanthropic Giumelli Family Foundation. F.L.M. receives salary support from Perron Institute. S.D.W. receives salary support from Murdoch Univesity and royalty payments from Sarepta Therapeutics from the sales of exon‐skipping drugs to treat DMD. P.A.A. is the Chief Scientific Officer of and receives salary support from Black Swan Pharmaceuticals (Wake Forest, NC, USA).

## Relevant conflicts of interest/financial disclosures

D.L. receives salary support from the philanthropic Giumelli Family Foundation. S.D.W. is a consultant to Sarepta Therapeutics and is named as an inventor on Duchenne muscular dystrophy exon‐skipping patents licensed through the University of Western Australia to Sarepta Therapeutics (Cambridge, MA, USA) and as such is entitled to milestone and royalty payments. P.A.A. is the Chief Scientific Officer of and receives salary support from Black Swan Pharmaceuticals (Wake Forest, NC, USA). Full financial disclosures and author roles may be found in the online version of this article.
